# From Mild Ataxia to Huntington Disease Phenocopy: The Multiple Faces of Spinocerebellar Ataxia 17

**DOI:** 10.1155/2014/643289

**Published:** 2014-10-02

**Authors:** Georgios Koutsis, Marios Panas, George P. Paraskevas, Anastasia M. Bougea, Athina Kladi, Georgia Karadima, Elisabeth Kapaki

**Affiliations:** ^1^Neurogenetics Unit, 1st Department of Neurology, University of Athens Medical School, Eginition Hospital, 74 Vas. Sophias Avenue, 11528 Athens, Greece; ^2^1st Department of Neurology, University of Athens Medical School, Eginition Hospital, 74 Vas. Sophias Avenue, 11528 Athens, Greece

## Abstract

*Introduction*. Spinocerebellar ataxia 17 (SCA 17) is a rare autosomal dominant cerebellar ataxia (ADCA) caused by a CAG/CAA expansion in the *TBP* gene, reported from a limited number of countries. It is a very heterogeneous ADCA characterized by ataxia, cognitive decline, psychiatric symptoms, and involuntary movements, with some patients presenting with Huntington disease (HD) phenocopies. The SCA 17 expansion is stable during parent-child transmission and intrafamilial phenotypic homogeneity has been reported. However, significant phenotypic variability within families has also been observed. *Report of the Family*. We presently report a Greek family with a pathological expansion of 54 repeats at the SCA 17 locus that displayed remarkable phenotypic variability. Among 3 affected members, one presented with HD phenocopy; one with progressive ataxia, dementia, chorea, dystonia, and seizures, and one with mild slowly progressive ataxia with minor cognitive and affective symptoms. *Conclusions*. This is the first family with SCA 17 identified in Greece and highlights the multiple faces of this rare disorder, even within the same family.

## 1. Introduction

Spinocerebellar ataxia 17 (SCA 17) is a rare form of autosomal dominant cerebellar ataxia (ADCA) caused by a coding CAG/CAA expansion in* TBP*, the gene for TATA-binding protein [1]. Fewer than 100 families have been described worldwide, from a limited number of countries [2, 3]. No cases from Greece have been reported to date.

SCA 17 is characterized by cerebellar ataxia, dementia, psychiatric features, and involuntary movements, primarily chorea and dystonia [4]. A wide variation in the clinical features of the disease has been noted, some patients having a typically ataxic phenotype and others having Huntington disease- (HD-) like picture [4]. In a few reports, phenotypic variability has been noted within the same family, despite the observed stability of the expansion during parent-child transmission [1, 5–7]. However, intrafamilial phenotypic homogeneity has also been emphasized in other cases [8].

We presently report the first family with SCA 17 from Greece, which was characterized by significant phenotypic heterogeneity regarding both disease presentation and severity.

## 2. Report of the Family

**Figure 1:**
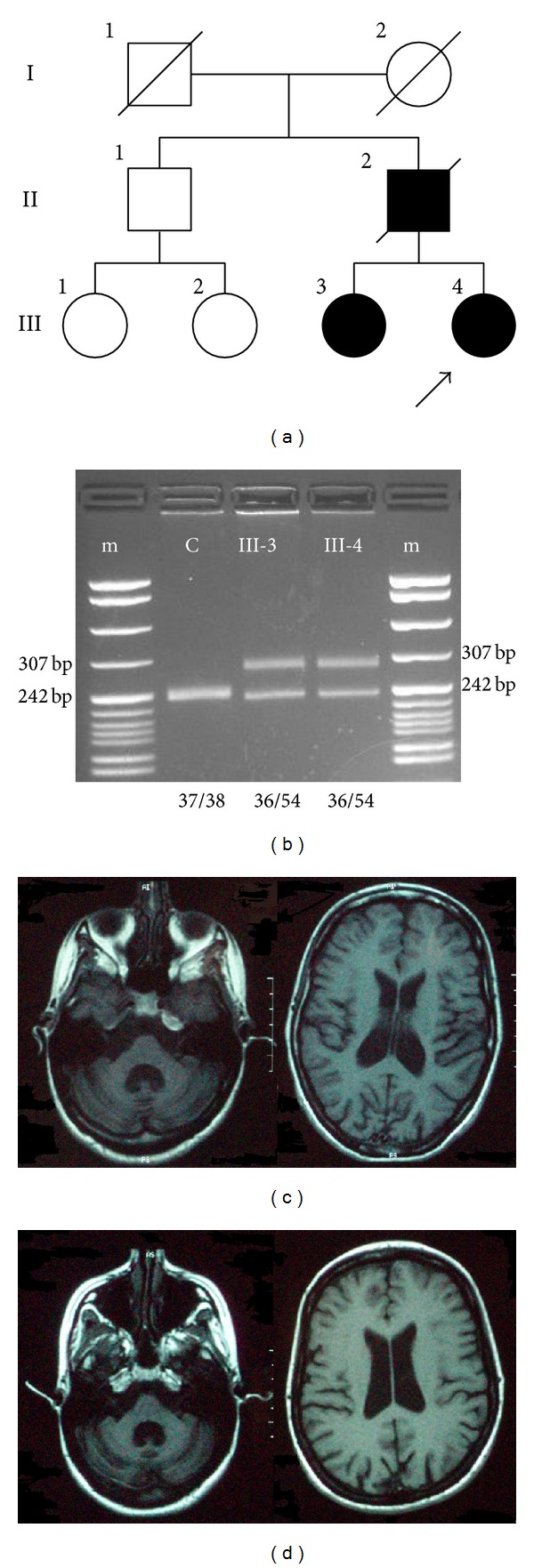
(a) Pedigree of the Greek family with a SCA 17 expansion. (b) PCR products of the two affected sisters and a normal control run with markers on a 4% agarose gel. (c) Brain MRI of case III-4 (index case) showing significant cerebellar atrophy and mild brainstem and cerebral atrophy. (d) Brain MRI of case III-3 showing significant cerebellar atrophy and mild brainstem atrophy.

The family was of Greek origin and included three affected members in two generations (Figure 1(a)). Grandparents died unaffected in their 8th and 9th decade. In cases III-3 and III-4, DNA was isolated from peripheral blood leucocytes following written informed consent. The CAG/CAA repeat of* TBP* was amplified by PCR using previously reported primers [5]. PCR products were checked on a 4% agarose gel (Figure 1(b)) and then run on an ABI310 genetic analyzer with a TAMRA 500 size standard.

## 3. Case III-4 (Index Case)

The proband was a 30-year-old woman who first developed progressive gait ataxia at the age of 25, which was subsequently followed by limb ataxia. Within three years she had significant cognitive impairment and had to stop working. Two years later she developed choreatic movements and more recently dystonia, followed by epileptic seizures. On examination she had a minimental score of 18/30 and a frontal assessment battery score of 11/18. She had severe constructional apraxia. There was moderate gait ataxia, dysmetria, dysdiadochokinesia, cerebellar dysarthria, and saccadic pursuit. Moderate chorea was present in the extremities. There was significant torticollis and limb dystonia. Asymmetric cogwheel rigidity was noted. Tendon reflexes were brisk but plantars were flexor. Brain MRI at disease duration of 3 years revealed cerebellar and mild brainstem and cerebral atrophy (Figure 1(c)). There were no evidence of peripheral nerve involvement on electrophysiological testing and no abnormalities on fundoscopic examination. Extensive investigations which included ceruloplasmin, acanthocytes, lipoproteins, hereditary metabolic screen, and genetic testing for HD, DRPLA, SCA1, SCA2, SCA3, SCA6, SCA7, SCA12, and FRDA were normal. Testing for the CAG/CAA expansion at the SCA 17 locus revealed a normal allele of 36 repeats and an expanded allele of 54 repeats (Figure 1(b)).

## 4. Case III-3

The 34-year-old sister of the proband had a much milder clinical picture, despite significantly longer disease duration. She first noticed mild gait ataxia at the age of 22, which progressed very slowly. Five years later she developed mild limb ataxia, which has also progressed slowly. More recently she has felt depressed and has noted minor memory problems. On examination she had a minimental score of 27/30 and a frontal assessment battery score of 16/18. There was mild gait ataxia, as well as mild dysmetria, dysdiadochokinesia, and cerebellar dysarthria. Very mild asymmetric cogwheel rigidity was also present. Tendon reflexes were normal and plantars were flexor. Brain MRI at disease duration of 8 years revealed cerebellar and mild brainstem atrophy (Figure 1(d)). Testing for the CAG/CAA expansion at the SCA 17 locus revealed a normal allele of 36 repeats and an expanded allele of 54 repeats (Figure 1(b)).

## 5. Case II-2

The father of the proband died at the age of 38, bedridden, and mute. He first developed gait unsteadiness and aggressive behavior at the age of 28. Within three years he developed frank psychosis with auditory and visual hallucinations and paranoid ideation. This was followed by cognitive impairment and choreoathetosis. At that stage he was diagnosed clinically as having HD. His condition progressed rapidly leading to death within 10 years from disease onset. He died before the advent of molecular testing and we have no data on the size of the SCA 17 expansion he carried.

## 6. Discussion

SCA 17 is a rare ADCA that has been reported most often from Japan, Germany, and Italy [1, 3, 6]. A few families have been reported from UK, France, Czech Republic, Taiwan, and India and single families have been reported from Belgium, USA, and Portugal [4, 9, 10]. Its minimal prevalence was estimated at 0.16/100,000 in the northeast of England [11]. In Greece it has never been reported to date and has not been detected in previous screens of patients with slowly progressive cerebellar ataxia or HD phenocopies [12, 13].

SCA 17 has been recognized as one of the most heterogeneous forms of ADCA with a wide clinical spectrum at presentation [4, 14]. The most common first symptom is ataxia, but cognitive decline, psychiatric symptoms, and chorea have also been frequently reported [1, 3, 5, 6]. As our family demonstrates, by the time patients present, they may have a relatively pure cerebellar ataxia, an ataxia with cognitive, psychiatric, and extrapyramidal features, or HD-like picture (hence the eponym HD-like 4 for SCA 17) [4]. Other features that have been reported in patients with SCA 17 include dystonia, parkinsonism, increased tendon reflexes, and seizures [6, 7, 14]. The absence of peripheral nerve involvement has also been noted and can be helpful in distinguishing from other ADCAs [3, 15]. Brain MRI usually reveals marked cerebellar atrophy, with milder cerebral atrophy and relative brainstem sparing [4]. Mild brainstem atrophy, as observed in the present cases, has also been occasionally reported [6, 16]. The degree of cerebral and cerebellar atrophy seems to correlate with the age of the patient and the size of the abnormal allele, and this has been recently confirmed by voxel-based morphometry [2, 17]. Genotype-phenotype correlations have been observed, with larger expansion sizes associated with earlier disease onset [4]. There is also some evidence that dystonia, increased deep tendon reflexes, and epilepsy are more common in patients with larger expansions in the range of 50–60 repeats [2, 6, 18].

The CAG/CAA repeat expansion in SCA 17 shows characteristic intergenerational stability that may be related to the presence of the CAA interruptions [3, 4, 6]. Thus, in most families all affected cases have identical expansions [3, 6]. This might be expected to lead to relative phenotypic homogeneity within families, as has indeed been reported in a family with SCA 17 and HD-like presentations [8]. However, in other rare cases where several affected family members have been examined, a broad intrafamilial phenotypic spectrum has been observed [1, 5–7]. The multiple faces of SCA 17, even within the same family, are clearly illustrated by our family, in which the father presented with HD phenocopy with rapid progression, the proband with progressive ataxia with dementia and involuntary movements, and her sister with a mild disorder consisting of a slowly progressive ataxia with minor cognitive and affective symptoms. Both sisters had an identical* TBP* expansion and their age at onset was relatively similar. Our findings reinforce the observation that repeat size has a limited influence on the course of pathology and the resulting severity of SCA 17 and that other modifying factors need to be taken into account [4].

In conclusion, the present communication highlights the multiple faces and remarkable intrafamilial heterogeneity displayed by SCA 17 and adds Greece to the limited number of countries with reports of this rare ADCA.
